# Supplementary material for: Ori-Finder 2022: A Comprehensive Web Server for Prediction and Analysis of Bacterial Replication Origins
Source: Genomics Proteomics Bioinformatics. 2022 Oct 17;20(6):1207–13. doi: 10.1016/j.gpb.2022.10.002 (PMC10225481; doi:10.1016/j.gpb.2022.10.002)
Supplement: Supplementary Table S2 [file mmc2.docx]

**Table S2 Indicator genes list for different chromosome type and lineage**

| **Chromosome type** | **Lineage** | **Indicator genes^1^** |
| --- | --- | --- |
| Main chromosome | Default | ***dnaA*** *dnaN rpmH gidA hemE mioC hemB* |
|  | Gammaproteobacteria | *dnaA dnaN rpmH* ***gidA*** *hemE mioC hemB* |
|  | Caulobacterales | *dnaA dnaN rpmH gidA* ***hemE*** *mioC hemB* |
|  | Rickettsiales | *dnaA dnaN rpmH gidA* ***hemE*** *mioC hemB* |
|  | Rhizobiales | *dnaA dnaN rpmH gidA* ***hemE*** *mioC hemB* |
|  | Cyannobacteria | *dnaA* ***dnaN*** *rpmH gidA hemE mioC hemB* |
|  | Chlamydia | *dnaA dnaN rpmH gidA hemE mioC* ***hemB*** |
| Secondary chromosome | Default | *repA repC parA parB dnaA* |

*Note*: ^1^, The indicator genes marked in bold below are principal indicator genes in the corresponding chromosome type or lineage, while others are secondary indicator genes.
